# Evaluation of maxillary arch symmetry in cleft patients undergoing orthodontic treatment: a comparative study

**DOI:** 10.1007/s00784-024-05656-9

**Published:** 2024-04-16

**Authors:** Maike Tabellion, Constanze Charlotte Linsenmann, Jörg Alexander Lisson

**Affiliations:** Department of Orthodontics (G56), Saarland University, Kirrberger Strasse 100, 66424 Homburg, Saar Germany

**Keywords:** Orthodontic treatment, Craniofacial anomalies, Cleft lip alveolus and palate, Facial asymmetry

## Abstract

**Objective:**

Patients with a cleft require structured procedures to achieve feasible treatment results. Since many treatment protocols coexist without being superior to one another, this study investigated the Saarland University Hospital treatment concept for patients with unilateral and bilateral clefts to evaluate its effects upon dental arch dimensions until the early mixed dentition.

**Material and methods:**

Digitized plaster models were used for data collection. Records of 83 patients (Cleft *n* = 41 [UCLP *n* = 28, BCLP *n* = 13], Non-Cleft Control *n* = 42) comprised 249 casts. The evaluation included established procedures for measurements of edentulous and dentate jaws. Statistics included Shapiro–Wilk, Friedmann, Wilcoxon and Mann–Whitney-U-Tests for the casts. The level of significance was set at *p* < 0.05.

**Results:**

The cast analysis showed an approximation of arch dimensions towards those of age-matched patients without a cleft until early mixed dentition. The mean values of patients with and without cleft lip and palate were almost indistinguishable when compared in primary and/or early mixed dentition.

**Conclusions:**

The evaluated treatment concept leads to feasible outcomes regarding dental arches in patients with unilateral and bilateral clefts compared to an age-matched non-cleft control.

**Clinical relevance:**

The evaluated treatment concept leads to favorable outcomes until early mixed dentition.

## Introduction

With an incidence of 1 out of 500 newborns, cleft lip and palate (CLP) represents the major percentage of craniofacial malformation with variable phenotypes [26]. It is the result of an incomplete or non-occurring fusion of maxillary and palatal parts between the 5th and 7th week of intrauterine life with a multifactorial etiology including genetic and environmental causes [14]. At this time, the embryonic development of the face and palate takes place [5]. Skeletal, functional, dental and aesthetic constraints are the consequence of this malformation [8]. Increased upper-jaw width and rotations of cleft segments are characteristic features for patients with a cleft [24]. Their rehabilitation requires structured procedures with a multidisciplinary approach, including orthodontics, maxillofacial surgery, pediatrics, genetics, otolaryngology, dentists and specialties of speech therapy to achieve treatment results that provide optimal function, stability and esthetics. Treatment starts soon after birth and continues up to adulthood [1, 2, 22]. Dimensional alterations of dental arches of patients with a cleft can influence the stability of the results gained during the individual rehabilitation. Therefore, the morphology and dimension of the upper arch and palate have been often investigated using two- or three-dimensional cast analyses [6] with landmarks of the gingiva [12, 13, 15, 16, 21, 23, 25, 27].

Studies investigating arch dimensions have been performed by Stancheva et al. [24] and included 50 patients with a unilateral cleft. They used a method described by Sillman [23] on two groups distinguished by one-step versus two-step surgical procedures with early treatment onset, and compared those to an age-matched non-cleft control. While patients of the early treatment group were more similar to the control group, both groups still showed significant differences of arch dimensions compared to the non-cleft control.

## Aims of the study

Since many treatment protocols coexist without being superior to one another, leaving the ideal treatment undetected, this study investigated the influence of the Saarland University Hospital Treatment Protocol for unilateral and bilateral clefts upon the outcome of upper arch dimensions until early mixed dentition. The use of landmarks on 3D digital casts should be verified as a useful method to study maxillary growth and development especially concerning effects of surgery and maxillary morphology alterations of patients with a cleft.

## Material and methods

### Patients

The study patients were divided into two groups (UCLP and BCLP), and compared to a non-syndromic and non-cleft age-matched control. Upper jaw casts of 41 non-syndromic patients (28 UCLP, 13 BCLP) with complete clefts at the age of 0–14 months were retrospectively identified and analyzed. All patients with a cleft were exclusively treated following the Saarland University Hospital CLP treatment concept.

The Saarland University Hospital Treatment Protocol (Table 1) includes the use of a Hotz-type plate [11] after birth and until palatoplasty, aiming at cleft width reduction with regard to plastic-reconstructive surgery. Four-week intervals were used to apply either abrasive adjustments of the palatal aspect or to renew the plate due to palatal growth. The morphological rehabilitation involves lip surgery with reconstruction of the nasal vestibule at the age of six months using Tennison-Randall technique [19]. This is followed by palatal surgery according to Widmaier-Cronin at the age of ten to twelve months, and reconstruction of the velar muscle sling up to six years according to Veau and Kriens [15]. Secondary alveolar bone graft follows between eight and ten years, depending on cleft adjacent tooth development and eruption. These surgical procedures are undertaken by oral- maxillofacial surgeons of the same University.

**Table 1:** Saarland University Hospital Treatment Protocol: Overview of Plastic reconstructive surgery and Orthodontics

Saarland University Hospital Treatment Protocol					
Plastic reconstructive surgery	6 months: closure of the lip + hard palate	10–12 months: closure of the soft palate	Up to 6 years: velopharyngoplastic surgery		Up to 10 years: osteoplastic surgery
	UCLP according to Tennison-Randall In the same intervention: closure of the hard palate according to Pichler	BCLP according to Veau-Manchester, simultaneously, plus perinasal reconstruction according to Delaire In the same intervention: closure of the hard palate according to Pichler	UCLP and BCLP according to Widmaier-Cronin	UCLP and BCLP according to Veau	Before eruption of permanent teeth when 1/2–2/3 of the root formation of the erupting lateral incisor or canine is finished
Orthodontics	After birth until closure of the soft palate			From the age of 5 years on	9–10 years
	Hotz-type plate			Early orthdontic treatment	Main treatment

### Inclusion/Exclusion criteria

The presence of complete cleft lip and palate with or without Simonart´s band and without previous surgery were the inclusion criteria. Exclusion criteria included comorbid syndromes, genetic disorders, Pierre Robin sequence and patients with an isolated cleft lip or palate. Furthermore, patients with transverse discrepancies (crossbite on one side or both sides) were included in the control group.

As a precondition, diagnostic data including of maxillary casts of certain intervals had to be present. Data were extracted from birth (t_0_), before (t_1_) and after (t_2_) the closure of the lip, after the closure of the palate (t_3_), which was the end of using the Hotz-type plate [11], and at the beginning of orthodontic treatment (t_4_) at the age of three to nine years. At t_4_ also lower jaw casts were analyzed.

### Control group

All patients with complete clefts (*n* = 41) were matched with casts of a non-cleft control (*n* = 42) aged between three and nine years. The control did not receive prior orthodontic treatment. Patients selected for the non-cleft control were otherwise healthy individuals who presented themselves for treatment of crossbites. 36 patients of the non-cleft control presented a unilateral crossbite (14 left sided, 22 right sided) and 6 patients a bilateral crossbite.

### Cast measurement

A total of 249 casts of patients with and without a cleft from one specialized center (University Hospital and Dental Medical School Homburg, Germany) were available. A subdivision by gender was not performed. The casts were digitized using a 3D scanner orthoXScan (orthoX®—DENTAURUM GmbH & Co. KG Ispringen, Germany). Angles and distances were measured on digital casts using the software OnyxCeph® 3TM (Image Instruments GmbH, Chemnitz, Germany) after programming an individual analysis.

### Landmarks and measuring technique

**Fig. 1:**
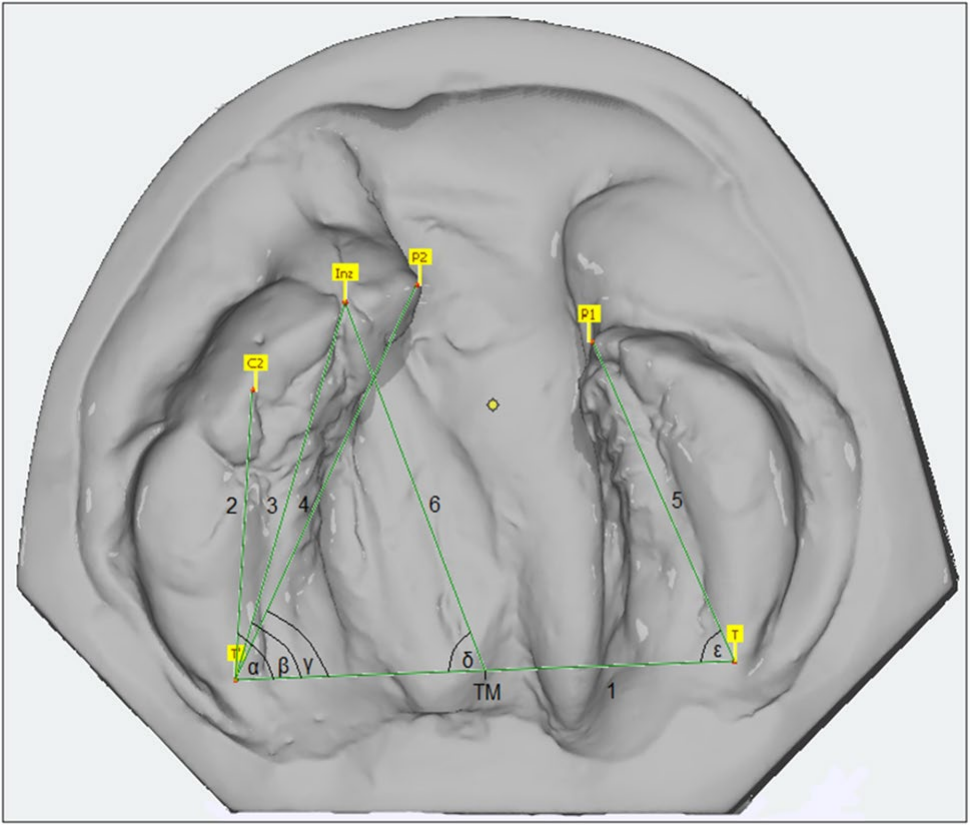
Overview of the landmarks used on the maxillary casts and the linear and angular parameters calculated from them for UCLP

**Fig. 2:**
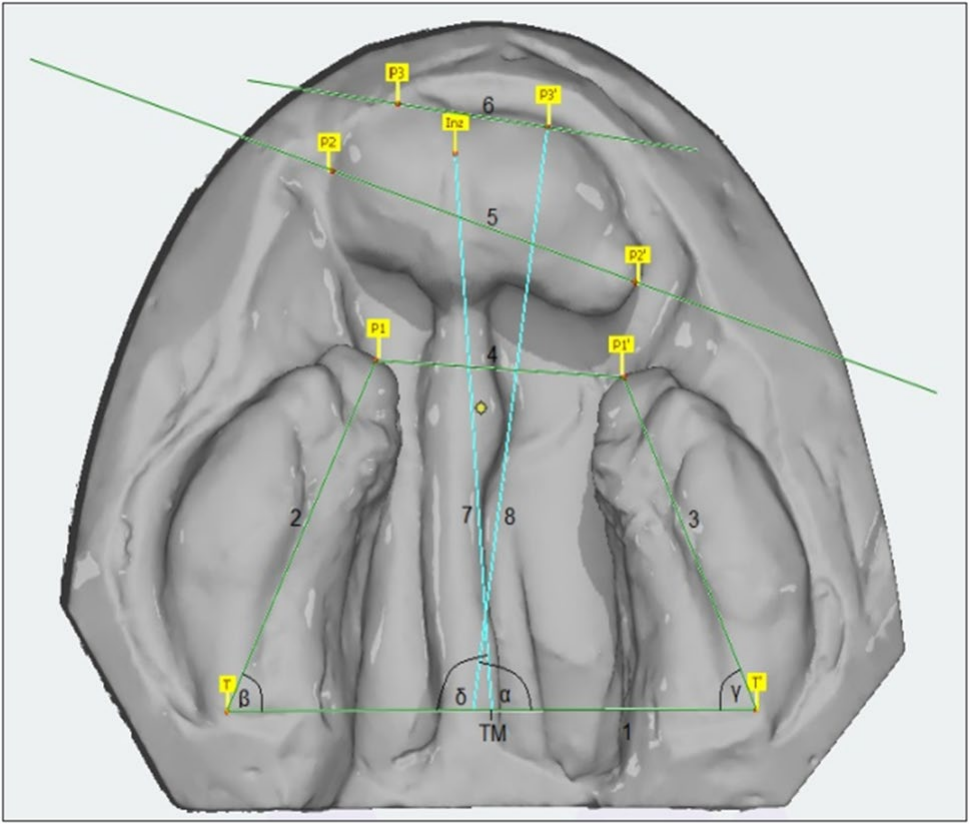
Overview of the landmarks used on the maxillary casts and the linear and angular parameters calculated from them for BCLP

The parameters for evaluation of the casts were based on anatomically defined landmarks (UCLP (Table 2) and BCLP (Table 3)) used by Lisson [17] and Hervatin and Köhler [10] for calculating distances and angles in all groups (UCLP (Fig. 1) and BCLP (Fig. 2)) between t_0_-t_3_.

**Table 2:** Landmarks, lines and measured angles and distances for UCLP

Constructed landmarks and lines	
TM	center of distance of the distal limits of the alveolar crests (tuberosity points, (T/T´))
Line 1	distance between landmarks T and T´ (base line)
Line 2	distance between landmark T´ and the point of the lateral sulcus (C2) distal of the canine germ
Line 3	distance between landmark T´ and Incisal point (Inz)
Line 4	distance between landmark T´ and anterior cleft edge point (P2)
Line 5	distance between landmark T and anterior cleft edge point (P1)
Line 6	distance between landmarks Inz and TM
Angles (°)	
α	angle between Line 1 and 2
β	angle between Line 1 and 3
γ	angle between Line 1 and 4
δ	angle between Line 1 and 6
ε	angle between Line 1 and 5

**Table 3:** Landmarks, lines and measured angles and distances for BCLP

Constructed landmarks and lines	
TM	center of distance of the distal limits of the alveolar crests (tuberosity points, (T/T´))
Line 1	distance between landmarks T and T´ (base line)
Line 2	distance between landmark T and anterior cleft edge point of the lateral segment (P1)
Line 3	distance between landmark T´ and anterior cleft edge point of the lateral segment (P1´)
Line 4	distance between landmark P1 and P1´
Line 5	secant between the lateral cleft edge points of the median segment (P2/P2´)
Line 6	tangent between the anterior cleft edge points of the median segment (P3/P3´)
Line 7	distance between incisal point (Inz) and TM
Line 8	reference line perpendicular to Line 6
Angles and distances	
α	angle between Line 1 and 8
β	angle between Line 1 and 2
γ	angle between Line 1 and 3
δ	angle between Line 1 and 7
ε	angle between Line 1 and 5
φ	angle between Line 1 and 6
Line 4 to 5	distance P1 to P2 and P1´ to P2´
LO	lead of P3 or P3´ to Line 4

For UCLP the angles α, β, γ and δ were used to evaluate the inclination of the large cleft segment while the angle ε was measured to describe the inclination of the small cleft segment in relation to the baseline. Adequate symmetry was achieved with an ideal angle δ of 90° and angles α and ε of equal size.

For BCLP the angles α, β, γ, δ, ε and φ were measured to describe the symmetry of the three cleft segments. Adequate symmetry was achieved with angles β and γ of equal size and the alignment of the median cleft segment perpendicular to base line. The ideal size of angles α and δ is 90°.

At t_4_, upper and lower anterior and posterior arch widths of patients with and without cleft were measured using primary molars or premolars and permanent molars.

### Statistical method, error of the method

Statistical analysis was performed with the SPSS software version 23 (IBM, Armonk, NY, USA). Normal distribution was tested with the Shapiro–Wilk test and visually. The Friedman and Wilcoxon-Test were used to analyze casts of UCLP and BCLP between t_0_-t_3_, the Mann–Whitney U test to compare casts of primary and mixed dentition at t_4_. The level of significance was set at *p* < 0.05. The significance level was defined as follows: *p* ≥ 0.05 not significant, *p* < 0.05 significant, *p* < 0.01 highly significant and *p* < 0.001 most highly significant. The effect size was tested by the formula *r* = Z/√N using Cohen´s criteria (for r): 0.1–0.3 = small effect size and low correlation, 0.3–0.5 = moderat effect size and correlation, > 0.5 = large effect size and high correlation.

For testing the intrarater-reliability the evaluation process was repeated on 20 randomly selected patients two months after the first investigation to evaluate the impact of landmarking errors, which involved removing and replacing the markings. The differences were statistically analyzed using Dahlberg´s error of the method (MF) with the formula MF = √(∑d^2^/2n), where *d* is the difference between two measurement results and *n* is the number of duplicate measurements [7]. The MF for angular and linear measurements in the present study was < 1 for all measurements.

## Results

### Case measurements

For UCLP (Table 4) the angles α, β, γ and ε showed highly significant decreases between t_0_-t_3_ (α: 84.29 ± 6.65° to 79.19 ± 5.37° (*p* = 0.007; *r* = 0.673); β: 65.79 ± 5.83° to 58.00 ± 5.81° (*p* = 0.001; r = 0.840); γ: 55.64 ± 5.29° to 43.94 ± 6.19° (*p* = 0.001; *r* = 0.853); ε: 69.86 ± 5.62° to 64.56 ± 5.23° (*p* = 0.014; *r* = 0.616)), δ a highly significant increase (76.29 ± 7.04° to 86.25 ± 4.33° (*p* = 0.001; *r* = 0.853)).

**Table 4:** UCLP cast analysis: changes of angles [°]. t_0_: age 1 week; t_1_: age 3.5 months; t_2_: age 9 months; t_3_: age 14 months, *M* Mean, *SD* standard deviation, ^a^Friedman- and Wilcoxon test of angles between t_0_-t_3_

Angles						
T	N	α	β	γ	δ	ε
		M ± SD	M ± SD	M ± SD	M ± SD	M ± SD
t_0_	28	84.29 ± 6.65	65.79 ± 5.83	55.64 ± 5.29	76.29 ± 7.04	69.86 ± 5.62
t_1_	26	85.04 ± 6.94	64.69 ± 6.44	52.46 ± 6.05	80.27 ± 5.65	67.93 ± 5.51
t_2_	26	81.39 ± 3.44	60.12 ± 4.74	46.58 ± 4.06	85.85 ± 4.76	67.27 ± 5.82
t_3_	16	79.19 ± 5.30	58.00 ± 5.81	43.94 ± 6.19	86.25 ± 4.33	64.56 ± 5.23
*P* value^a^ t_0_-t_1_		0.587	0.311	0.013	0.002	0.023
*P* value^a^ t_0_-t_2_		0.028	0.000	0.000	0.000	0.100
*P* value^a^ t_0_-t_3_		0.007	0.001	0.001	0.001	0.014
*P* value^a^ t_1_-t_2_		0.010	0.001	0.000	0.001	0.552
*P* value^a^ t_1_-t_3_		0.005	0.001	0.001	0.001	0.140
*P* value^a^ t_2_-t_3_		0.126	0.060	0.083	0.324	0.312

The large cleft segment moved towards the midline and its anterior cleft edge point was located in a dorsal and lateral way. The small cleft segment remained unchanged.

For BCLP (Table 5) the angle γ and the distance P1-P1´ decreased significantly between t_0_-t_3_ (γ: 74.82 ± 5.72° to 70.47 ± 5.27° (*p* = 0.043; *r* = 0.826); P1-P1´: 22.13 ± 3.31 mm to 17.62 ± 3.97 mm (*p* = 0.043; *r* = 0.826)), while P2-P2´ increased (17.35 ± 4.02 mm to 17.83 ± 2.06 mm (*p* = 0.043; *r* = 0.826)). α and φ showed a significant decrease between t_0_-t_1_ (α: 98.07 ± 17.17° to 90.07 ± 13.15° (*p* = 0.016; *r* = 0.693); φ: 8.07 ± 17.17° to 2.60 ± 12.87° (*p* = 0.016; *r* = 0.693)). The distance LO (P3/P3´ perpendicular to P1-P1´) decreased highly significant between t_0_-t_2_ (12.36 ± 4.07 mm to 9.07 ± 3.87 mm (*p* = 0.006; *r* = 0.765)). The angles β, δ, ε showed no significant changes (*p* ≥ 0.05). After the closure of the soft palate (t_3_) ε could not be measured any more. The lateral cleft segments approximated to the medial cleft segment. The medial cleft segment was aligned with the base line.

**Table 5:** BCLP cast analysis: changes of angles [°] and distances [mm]. t_0_: age 1 week; t_1_: age 3.5 months; t_2_: age 9 months; t_3_: age 14 months, *M* Mean, *SD* standard deviation, ^a^Friedman- and Wilcoxon test of angles and distances between t_0_-t_3_

Angles							
T	N	α	β	γ	δ	ε	φ
		M ± SD	M ± SD	M ± SD	M ± SD	M ± SD	M ± SD
t_0_	13	98.07 ± 17.17	74.49 ± 5.56	74.82 ± 5.72	88.32 ± 12.46	9.75 ± 18.00	8.07 ± 17.17
t_1_	12	90.07 ± 13.15	73.64 ± 4.29	75.32 ± 3.57	90.81 ± 11.72	5.36 ± 14.01	2.60 ± 12.87
t_2_	14	88.81 ± 11.67	72.08 ± 6.18	72.76 ± 5.83	89.15 ± 6.77	3.19 ± 9.55	0.71 ± 11.71
t_3_	6	92.90 ± 12.10	73.62 ± 6.69	70.47 ± 5.27	87.67 ± 8.66	-	8.70 ± 8.11
* P* value^a^ t_0_-t_1_		0.016	0.657	0.722	0.248	0.155	0.016
* P* value^a^ t_0_-t_2_		0.075	0.345	0.173	0.289	0.133	0.075
* P* value^a^ t_0_-t_3_		0.138	0.893	0.043	0.686	-	0.138
* P* value^a^ t_1_-t_2_		0.844	0.530	0.272	0.477	0.937	1.000
* P* value^a^ t_1_-t_3_		0.833	0.345	0.046	0.600	-	0.599
* P* value^a^ t_2_-t_3_		0.753	0.917	0.046	0.600	-	0.917
Distances							
T	N	P1-P1´		P2-P2´		LO	
		M ± SD		M ± SD		M ± SD	
t_0_	13	22.13 ± 3.31		17.35 ± 4.02		12.36 ± 4.07	
t_1_	12	20.20 ± 2.83		18.14 ± 3.90		12.57 ± 3.82	
t_2_	14	19.85 ± 3.73		19.46 ± 4.16		9.07 ± 3.87	
t_3_	6	17.62 ± 3.97		17.83 ± 2.06		10.67 ± 3.08	
* P* value^a^ t_0_-t_1_		0.050		0.016		0.333	
* P* value^a^ t_0_-t_2_		0.055		0.009		0.006	
* P* value^a^ t_0_-t_3_		0.043		0.043		0.138	
* P* value^a^ t_1_-t_2_		0.875		0.050		0.002	
* P* value^a^ t_1_-t_3_		0.028		0.046		0.916	
* P* value^a^ t_2_-t_3_		0.058		0.500		0.027	

At t_4_ (Table 6), upper and lower anterior (UA, LA) and posterior (UP, LP) arch widths (AW) of patients with cleft lip and palate were not different from a non-cleft control during primary dentition (UAAW: 20.05 ± 2.20 mm versus 21.67 ± 1.51 mm; UPAW: 29.64 ± 2.95 mm versus 29.00 ± 1.90 mm; LAAW: 18.41 ± 1.56 mm versus 18.83 ± 1.33 mm; LPAW: 28.64 ± 1.92 mm versus 29.00 ± 2.10 mm; *p* ≥ 0.05) and mostly during mixed dentition (UAAW: 32.88 ± 3.68 mm versus 35.12 ± 1.87 mm; LAAW: 35.17 ± 0.75 mm versus 35.22 ± 2.37 mm; LPAW: 47.63 ± 3.38 mm versus 47.39 ± 3.11 mm; *p* ≥ 0.05). The upper posterior arch width was significantly smaller for non-cleft control during mixed dentition (UPAW: 46.88 ± 4.05 mm vs. 43.33 ± 2.61 mm (*p* = 0.022; *r* = 0.380)).

**Table 6:** Upper and lower anterior and posterior arch widths [mm] during t_4._ t_4_ pretreatment visit, *M* Mean, *SD* standard deviation, ^a^Mann-Whitney U test between groups at t_4_

	Cleft group (*N* = 21) M ± SD	Non-cleft control (*N* = 6) M ± SD	*P* value^a^
Primary dentition			
UAAW	20.05 ± 2.20	21.67 ± 1.51	0.057
UPAW	29.64 ± 2.95	29.00 ± 1.90	0.566
LAAW	18.41 ± 1.56	18.83 ± 1.33	0.643
LPAW	28.64 ± 1.92	29.00 ± 2.10	0.643
	Cleft group (*N* = 8) M ± SD	Non-cleft control (*N* = 18) M ± SD	*P* value^a^
Mixed dentition			
UAAW	32.88 ± 3.68	35.12 ± 1.87	0.066
UPAW	46.88 ± 4.05	43.33 ± 2.61	0.022
LAAW	35.17 ± 0.75	35.22 ± 2.37	0.626
LPAW	47.63 ± 3.38	47.39 ± 3.11	0.935

The cast analysis showed an approximation of arch dimensions towards those of age-matched patients without a cleft until early mixed dentition. The mean values of patients with and without cleft lip and palate were almost indistinguishable when compared in primary and/or early mixed dentition.

## Discussion

### Study patients and control

Digitized plaster models of patients with and without cleft were analyzed. The patients with a cleft received their entire treatment exclusively at University Hospital and Dental Medical School Saarland. Exclusion criteria included comorbid syndromes, genetic disorders, Pierre Robin sequence and patients with an isolated cleft lip or palate. A gender division was not performed due to the number of participants. The total patient figures for this study were, however, acceptable. Still, after division into different groups, necessary for comparison of patients of the same age, the number of patients per group remained low. Lower jaws of patients with a cleft have not been analyzed, because quite contrary to the upper jaws notable deficits were not remarkable. The numerous different treatment protocols in existence did not allow the inclusion of casts from other centers in order to increase numbers. However, coordinated procedures with a multidisciplinary approach starting with orthodontic treatment using a Hotz-type plate [4, 9, 11] soon after birth appear to be useful for feasible treatment outcomes. Mishima et al. [20] described the incorporation of a plate as a guarantee of reaching maxillary symmetry.

The suitability of the control is only partially given. The controls were age-matched, had no cleft and were thus presenting a normal arch symmetry. Still, the controls could only be acquired because they presented themselves for early orthodontic treatment. This again was justified due to transverse deficiencies and probably some form of maxillary micrognathia. Therefore, it was possible to gain evidence about effects upon arch shape and symmetry. It was, however, impossible to judge further skeletal parameters at this point, for this requires a radiographic comparison for which no justifying indication existed at the point of examination.

### Cast analysis

Systematic and continuous documentation of patients with a cleft should be emphasized, because the rehabilitative treatment is a challenging long-term procedure. Evaluation procedures starting shortly after birth are crucial when intending to compare treatment protocols and outcomes. Dental casts or – if possible – 3D scans thus represent an important documentation tool. Arch dimension and landmark analyses have proven useful to compare treatment outcomes, even though landmarks are easier to find on casts of non-cleft patients than on those of patients with a cleft. Studies of maxillary dimensions of patients with a cleft lead to better understanding of morphological changes [6, 12, 13, 15, 16, 21, 23–25, 27, 28].

Dimensional and positional changes of dental arches of patients with a cleft can influence the stability of the results gained during rehabilitation of the individual. This led to frequent investigations of maxillary morphology and dimension through two- or three-dimensional cast analyses [6] with landmarks of the gingiva [12, 13, 15, 16, 21, 23, 25, 27]. Stancheva et al. [24] had a total of 204 maxillary casts for a three-dimensional analysis of maxillary development in 50 UCLP patients during the first six years of life. The measurements were performed with a Reflex Microscope from birth up to 71 months of age and based on anatomically defined landmarks described by Sillmann [23] amongst others, calculating distances and angles. The patients were divided into two treatment groups. The early treatment group underwent two-stage cleft closure with single-stage palatoplasty at ten to fourteen months. The late treatment group had a two-stage cleft closure with palatoplasty at four to seven years. Until palatoplasty, the patients were treated using a Hotz-type plate [11]. The control group included 17 patients without a cleft. Patients of the early treatment group were more similar to the control group. Other than the patients of this investigation, both groups (early and late treatment) showed significant differences concerning both shape and arch widths compared to the control group.

Athanasiou et al. [3] found differences in arches of patients with UCLP. Dental casts were analyzed during deciduous, mixed and permanent dentition. The dental arches were significantly narrower and shorter than age-matched arches without cleft. Until the age of 12 years, however, arch symmetry and dimensions were improved towards nearly equal values as found in a non-cleft age-matched control. Maxillary arch length was always more affected than width. An increased presence of crossbites and underdevelopment of the maxillary length were results from decreased maxillary growth. These differences are possibly caused by a higher quantity of participants of the study, lack of data and effects of the initial cleft size and three different surgeons performing the operations.

Melissaratou and Friede [18] found differences in arches and occlusion of patients with BCLP after two different routines for palatal surgery. Early two-stage palatal closure was performed during the first year of life or delayed hart palate repair at around eight years with soft palate closure at twelve months of age. All surgeries were performed by the same surgeons. They compared long-term results of maxillary morphology, dental arches and occlusion with respect of the timing of hard palate repair, but in favor of delayed procedures. The overbite, overjet and crossbite scores at three years of age were significantly better in the group of delayed hart palate repair. In addition, the crossbite score at ten years of age was better for these patients.

## Conclusions

The evaluated treatment concept leads to favorable outcomes until early mixed dentition with regard to dental arch symmetry in patients with unilateral and bilateral clefts when compared to an age-matched non-cleft control. The upper arch symmetry of patients with a cleft improved considerably until then. At early mixed dentition, mean differences between patients with and without cleft remain neglectable. However, patients with a cleft were compared to a non-cleft control presenting crossbites in the transverse dimension, which could be again caused by restricted transverse maxillary growth.

The investigated treatment concept appears to reduce unwanted side effects of cleft rehabilitation, i.e., maxillary growth restriction or collapsing of the cleft segments. Larger patient numbers, however, are necessary for a final assessment.

## Data Availability

Not applicable.
